# Physical fitness and motor competence performance characteristics of Chinese elite youth athletes from four track and field throwing disciplines—a cross-sectional study

**DOI:** 10.3389/fphys.2023.1267804

**Published:** 2023-12-15

**Authors:** Kewei Zhao, Maximilian Siener, Yifan Zhao, Andreas Hohmann

**Affiliations:** ^1^ High Performance Research Center, China Institute of Sport Science, Beijing, China; ^2^ BaySpo—Bayreuth Center of Sport Science, University of Bayreuth, Bayreuth, Germany; ^3^ Institute of Professional Sport Education and Sport Qualifications, German Sport University Cologne, Cologne, Germany

**Keywords:** shot put, hammer throw, discus throw, javelin throw, discriminant analysis, multilayer perceptron

## Abstract

**Purpose:** For systematic athletic training and targeted talent development, it is essential to know the physical fitness and motor competencies of top athletes in detail. However, it can be difficult to identify differences in performance requirements and thus to provide adequate support, especially for sports that at first glance appear to have similar demands—such as track and field throwing disciplines. Therefore, the aim of the study was to examine the physical fitness and motor competence of top athletes from different throwing disciplines and to check whether the athletes’ performance parameters match the specific requirements of the respective sport.

**Methods:** The study involved 289 male youth athletes (aged 14–18 years) across four distinct throwing disciplines: shot put (*n* = 101), hammer throw (*n* = 16), discus throw (*n* = 63), and javelin throw (*n* = 109). The performance evaluation comprised three anthropometric measurements and twelve motor performance prerequisites applicable to the throwing disciplines. Discriminant analysis and neural networks (Multilayer Perceptron) were implemented to determine the possibility of distinguishing among athletes from the four sports.

**Results:** The study’s findings indicate that in male throwing athletes, disparities in general physical fitness and motor proficiency assessments discern the majority of talented young athletes based on their specific sport (discriminant analysis: 68.2%; multilayer perceptron analysis: 72.2%). This remains applicable irrespective of the classification method employed. Discus throwers possessed a height advantage, while shot putters and hammer throwers exhibited superior arm strength. Javelin throwers displayed better explosive strength and sprinting speed. Except for the hammer throwers, all events demonstrated a high level of explosive power in the medicine ball forward or backward throw test, which was especially crucial for shot put and discus athletes.

**Conclusion:** The significance of physical fitness and motor competence tests in identifying and transferring talented athletes in track and field throwing disciplines has been affirmed. Using linear and non-linear classification methods, most athletes could be assigned to their correct sport. However, this also shows that slightly different training and talent identification is required for each of these sports. Furthermore, non-linear analysis methods can provide useful support for the development processes in junior competitive sports.

## Introduction

**FIGURE 1:**
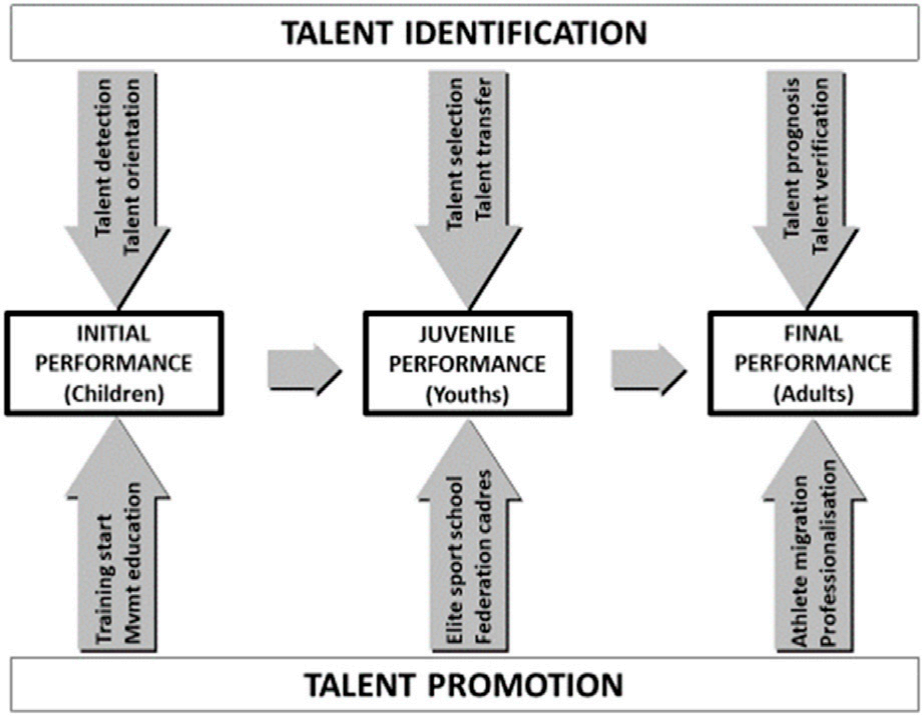
Talent identification and talent promotion as the two intertwined strategies in the long-term talent development model (mod. from Hohmann and Seidel, 2003).

Participation in elite sport training at a young age is associated with identification, selection, and also transfer of athletes (Figure 1) with specific performance prerequisites of a particular sport (Baker et al., 2012; Cobley et al., 2012; Pion, 2015). In Line with the talent development model of Preckel et al. (2020), athletes in middle and late adolescence tend towards late specialisation, which is particularly true for youth in the throwing events. Therefore, coaches and applied sports scientists try to identify or transfer talented throwers from the athletic population, based on characteristics of physical fitness and motor competence, which are assumed to be important for future success at a high level (Siener and Hohmann, 2019). Due to the specific nature of the throwing motion, the performance requirements differ significantly from those of other athletic disciplines, which highlights the need for specific studies on the relevant components of physical fitness and motor competence (Zhao and Zhao, 2023). For instance, certain anthropometric characteristics are exhibited by athletes in shot put, hammer, and discus throw, including a higher body weight and lean body mass when compared to javelin throw athletes (Zaras et al., 2021). Furthermore, shot put and discus throw athletes usually possess a superior body height in contrast to javelin throwers, as found by Carter. (1982) and Morrow et al. (1982).

In the realm of physical fitness, athletes in all four throwing disciplines rely on maximum arm and leg strength and ballistic power of the extremities to enhance their performance. The strength of arm and leg extension muscles is crucial in achieving a high momentum transfer to the throwing device, making these performance characteristics pivotal (Łysoń-Uklańska et al., 2021). Terzis et al. (2003) and Bouhlel et al. (2007) discovered that maximal leg and arm strength were significantly correlated with throwing performance in shot-put and javelin throw, respectively. Moreover, this crucial connection has been presented not only in similarly diverse athletes at the university level (Stone et al., 2003; Zaras et al., 2013), but also at the relatively homogeneous elite performance level (Schleichardt et al., 2021). Research conducted by Caughey and Thomas (2022) on collegiate shot put athletes has shown that sprinting speed also has a significant impact. Explosiveness is not the only factor that affects throwing performance. Additionally, the core stability (Okada et al., 2011; Jha et al., 2022) and flexibility (Kim et al., 2014) of athletes play a crucial role in throwing disciplines, not only in the shoulder but also in the trunk, hip and legs. Although no reports exist on the validity of running endurance tests in track and field throwing disciplines, these tests, conducted using essential exercise modes, are frequent in seasonal preparation phases for all four throwing disciplines. Therefore, it appears reasonable to assess and examine endurance capabilities.

The identified sport-specific performance characteristics of shot put, hammer throw, discus throw, and javelin throw in track and field can be used for talent identification or transfer during middle and late adolescence, based on the aforementioned research (Collins et al., 2014; MacNamara and Collins, 2015). Horst et al.’s (2020) findings highlight the importance of exploring performance similarities and differences across the four throwing disciplines. Identical patterns of individual performance characteristics were observed among shot put, discus, and javelin decathlon athletes. Therefore, investigating both similarities and differences is necessary in this context.

Athletic abilities in specific sports adhere to a combination of inherent talents (nature) and cultivated performance prerequisites (nurture; Pion, 2015). Talent identification protocols involve both morphological measurements and recorded performance tests. Therefore, several talent identification and transfer programs at elite sports schools have incorporated morphological, physical fitness, and motor competence diagnostics (Hoare, 1995; Fuchslocher et al., 2011; Unnithan et al., 2012; Douglas, 2014; Fernandez-Fernández et al., 2014).

Many sports rely on a complex, multidimensional performance profile (Buekers et al., 2015). As such, the selection of talented athletes should emphasise a multifaceted range of general physical, physiological, psychomotor and psychological performance diagnostics (Williams and Franks, 1998; Williams and Reilly, 2000). In general, there is a lack of research that investigates the discriminatory value of various performance prerequisites across a range of different sporting disciplines. However, studies have shown potential in distinguishing sports based on their performance requirements. For instance, Leone et al. (2002) successfully differentiated 88% of athletes from four sports (figure skating, swimming, tennis, and volleyball) by conducting a discriminant analysis that factored in athletes’ anthropometric and motor traits. Opstoel et al. (2015) reported an accurate classification of 85.2% of highly active U12 athletes across various sports such as ball sports, dance, gymnastics, martial arts, racket sports, and swimming. Furthermore, Pion et al. (2015) achieved a successful classification of 96.4% of 141 adolescent Flemish athletes across nine distinct sports. In accordance with our research objectives, the results from Pion et al.'s (2014) investigation into elite male U18 athletes were highly favourable, with a 100% accurate classification achieved for the interconnected martial arts disciplines of judo, karate, and taekwondo. Unlike the studies mentioned earlier, the accuracy of discriminant analysis reduces when one case (n = 1) is held out to classify on the basis of discriminant functions obtained from all other cases (n–1), unless the hold-out case is representative of the entire population under investigation. Zhao et al. (2019a) employed this cross-validation approach to discriminate 56 youth athletes aged 12–16 years from sports such as basketball, fencing, judo, swimming, table tennis, and volleyball. The success rate for the correct classification of athletes was reported to be 71.3%. Applying a 10% holdout strategy and utilising the multilayer perceptron (MLP) neural network method as an alternative, the authors reported a classification rate of 71.0%, which was almost identical.

To suggest appropriate sports for young athletes based on their unique talent profile, which is feasible during early childhood (Pion et al., 2015) and is also a part of a talent transfer strategy at later stages (Bullock et al., 2009), trustworthy and reliable data on the potential of gifted athletes in particular sports is critical for applied sports scientists. However, multifaceted test batteries that can distinguish between the relevant performance attributes necessary for different sports disciplines are also needed in clubs and sports federations. Thus, the present study aims to distinguish elite male athletes from four distinct track and field throwing disciplines using physical fitness and motor competence tests. Furthermore, it will explore whether the athletes from each discipline possess a sport-specific anthropometric and motor performance profile that aligns with the unique demands of their throwing discipline. While top-level performance components at a young age might not be essential prerequisites for future success (Boccia et al., 2019; Boccia et al., 2021), assessments of morphological, physical fitness and motor competence can aid in identifying or transferring youth athletes into specific sports or sport-specific disciplines (Collins et al., 2014). Although the development and success of elite athletes are influenced by various complex and dynamically changing factors (Hartigh et al., 2016), such as the different rules and competition devices for youth throwing athletes, it seems feasible to orientate young athletes towards appropriate sports based on their core physical fitness and motor competence attributes during their formative years. Zhao and Zhao (2023) found significant correlations between linear sprint speed, lower and upper limb power, and performance in the four track and field throwing disciplines. For example, the overhead medicine ball forward and backward throw had a particularly strong correlation. Additionally, male and female youth athletes differed in body height, body mass, and BMI, as well as in trunk flexibility, core stability, and performance on the hexagon agility test. It was hypothesized that a generic test battery would have enough discriminative power to categorise athletes into their respective sports, based on their unique profile of test results. The presiding athletic performance prerequisites could therefore serve as a scientific foundation for identifying or transferring talented athletes in this field of sport.

## Materials and methods

### General study design

Chinese male junior athletes from four different track and field throwing disciplines underwent a generic test battery to evaluate their physical fitness and motor competence. Additionally, body height and weight measurements were obtained from each participant. The study aims to investigate the direct correlation between the test results and the achieved throwing performances. Furthermore, using both linear and non-linear classification analyses, we explored the potential for differentiating between sport disciplines based on test values.

### Participants

A cohort of N = 348 young male athletes from elite track and field programs throughout China were chosen as subjects for this study. The sports schools they attended were among China’s thirty most prestigious. The selected athletes, aged 14–18 years, specialised in one of four throwing disciplines: shot put (n = 155), hammer throw (n = 35), discus throw (n = 116), and javelin throw (n = 148). All athletes had previously competed in either provincial or national junior athletic championships. All athletes engaged in a minimum of two daily training sessions, totaling 18 h of training per week over six training days. The athletes competed at a high level in their respective sports, representing China or one of its 22 provinces at nationwide competitions. Participant recruitment occurred in adherence to ethical standards set by the China Institute of Sport Science (CISS) and the Chinese Athletics Association (CAA). The Chinese Athletics Association (CAA) organized the test battery and executed the tests. The data utilized in this study, as presented in Table 1, was diagnosed during the first month (September/October) of the 2022/2023 training year.

**TABLE 1:** Descriptives of the specific throw performances, three morphological and twelve physical fitness and motor competence characteristics.

	N	Mean	SD	SE	Min	Max
Age	348	15.93	1.01	0.05	13.83	17.75
Shot put standing (m)	155	13.44	2.69	0.22	6.18	18.26
Hammer throw spinning (m)	35	37.42	15.41	2.60	10.00	60.00
Discus throw standing (m)	116	40.67	7.31	0.68	16.00	56.00
Javelin throw standing (m)	148	36.70	7.17	0.59	15.00	50.00
Body height (cm)	306	181.63	6.53	0.37	166.00	200.00
Body weight (kg)	306	88.31	18.42	1.05	52.00	145.00
BMI	348	26.65	5.20	0.28	10.00	43.20
Standing long jump (m)	348	2.55	0.27	0.01	1.40	3.22
Triple jump (m)	340	7.67	1.18	0.06	4.90	12.34
Pull-ups (n)	348	8.90	7.44	0.40	0.00	43.00
Plank (s)	348	159.28	73.97	3.97	39.00	660.00
Sit and reach (cm)	348	17.14	7.04	0.38	0.00	47.00
30-m sprint (s)	347	4.39	0.42	0.02	3.69	6.00
60-m sprint (s)	339	8.13	0.77	0.04	6.60	11.39
Medicine ball throw forward (m)	348	13.08	2.38	0.13	5.00	20.55
Medicine ball throw backward (m)	340	15.05	3.25	0.18	5.00	21.80
Hexagonal agility run (3 reps; s)	348	15.92	8.17	0.44	9.00	89.00
Balance pad one-leg stance (s)	314	50.93	22.69	1.28	10.00	186.00
2-km run (min)	348	9.93	1.56	0.08	6.25	16.34

Ethics approval and parental written informed consent was obtained from the participants in this study in accordance with the declaration of Helsinki. Parents of all athletes were informed of the study protocol, which was outlined in an information letter. No data collection took place without parents’ consent. All athletes performed at a high level in their respective sport and represented China and/or one of the 22 provinces in provincial or national competitions.

### Measurements

The participants underwent two morphological, nine physical fitness tests, and three motor competence tests. All tests were carried out by sports school staff of over twenty experts in sports science and coaching. This staff received pretesting training to standardize the testing procedures (Zhao and Zhao, 2023). The experimenters testing the athletes remained the same, ensuring that measurement consistency was achieved. All tests were conducted on the initial day with the Chief Judge being video-linked to the referees at each test location to commence each event in unison, following the test’s content. The tests began at 9 a.m., and all athletes refrained from strenuous exercise the day before the test session.

**FIGURE 2:**
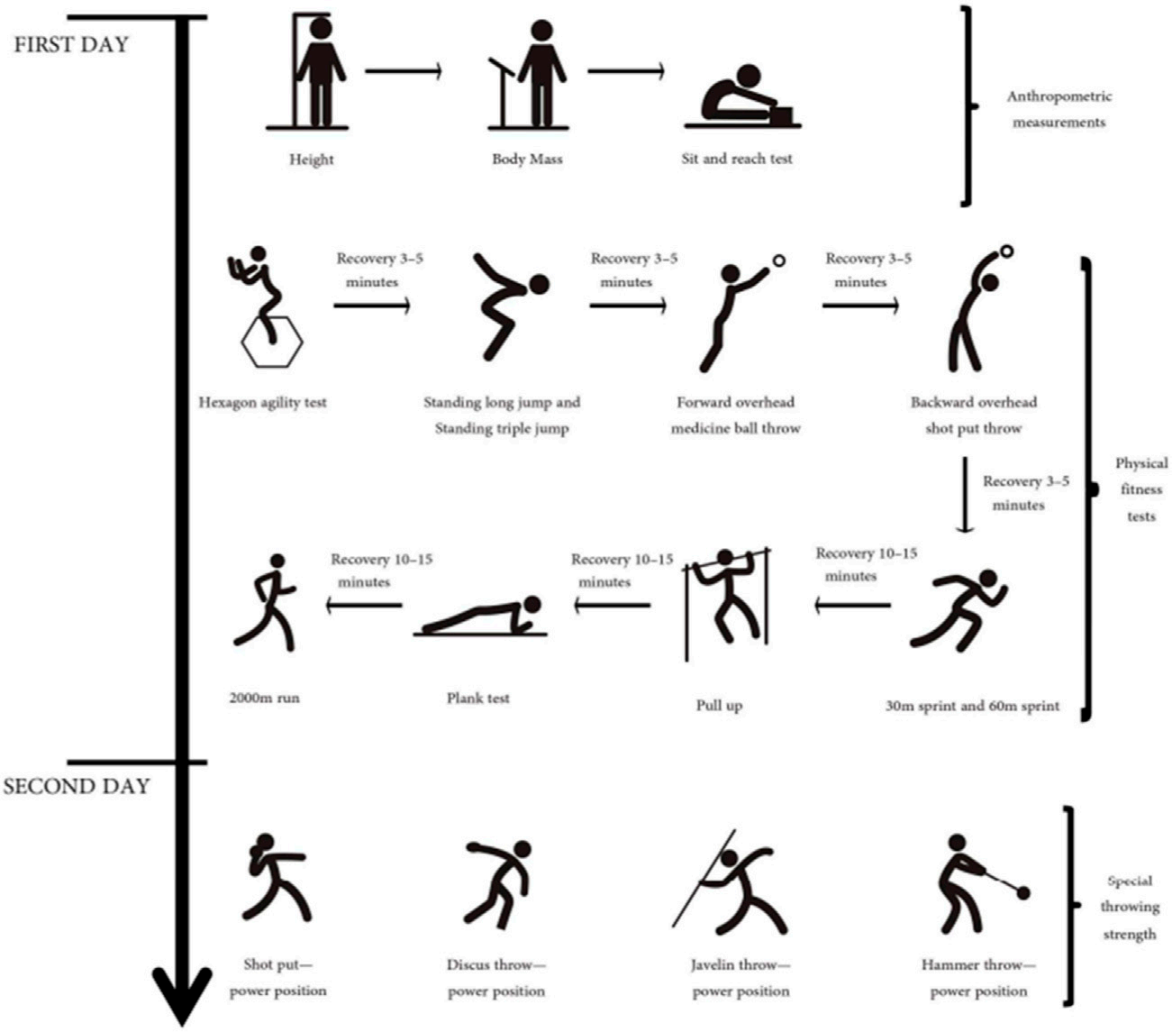
Test order on day 1 and order of the specialized throwing performance assessment on day 2 (Zhao and Zhao, 2023).

The measurements and tests were executed by each participant before standardized training and explanations were provided. All participants adhered to the prescribed warm-up procedure, comprising jogging, dynamic stretching, activation, and potentiation techniques. Anthropometric measurements were taken in the morning of the first day, followed by physical fitness testing in the afternoon. Figure 2 illustrates the test order to ensure that the completion of one test did not impede performance in subsequent tests. Specialized throwing performance was then assessed on the second morning. The results of all athletes’ tests were recorded in accordance with each test’s specific requirements.

#### Morphological characteristics

The subjects followed a standardized procedure for measuring body height (BH) to the nearest 0.1 cm (Height Tester, Donghuateng Sports Apparatus Ltd., Beijing, China). Also, body weight (BW) was measured according to standardized test procedures to the nearest 0.1 kg (calibrated Seca Alpha 770; Norton, 2018). Body Mass Index (BMI) of each athlete was calculated by dividing their weight by the square of their height (kg/m^2^). As in some cases only the BMI was reported instead of the BH and BW data—in most of the analyses the BMI was used.

#### Motor characteristics

The participants’ physical fitness (PF) was evaluated through nine generic test procedures (Zhao and Zhao, 2023). The PF assessment battery assessed explosive leg strength (measured via two attempts of a standing long jump and triple step jump), arm strength (measured through chin-over-the-bar pull-ups and two attempts of a medicine ball throw backwards and forwards; refer to Table 2), core strength (measured via the plank test), endurance (measured by a 2 km run), and running speed (measured via single attempts of both the 30 m and 60 m running sprint).

(1) Standing Long Jump and Standing Triple Jump

**TABLE 2:** Weights of the test devices (Zhao and Zhao, 2023).

Test	Under 16 Years (kg)	Under 18 Years (kg)
MBFT	4.0	5.0
MBBT	4.0	5.0
Shot put	4.0	5.0
Hammer throw	4.0	5.0
Discus throw	1.0	1.5
Javelin throw	0.6	0.7

Several studies have shown that the standing long jump (Siener et al., 2021b) and standing triple jump can effectively assess lower limb strength and power, as well as being highly correlated with the anaerobic output capacity of young individuals. Participants were given instruction to wear sneakers while standing behind the jumping line on a running track and then jump forward as fast as possible with an arm swing. All participants undertook three long jump and three triple jump trials, beginning from the edge of the jump line with 1-min rest between each attempt. Standardized guidelines were issued before the test. The gap between the jump line and the nearest contact point of the body with the ground was measured to the closest unit of 1 cm. The farthest distance was documented.

(2) Pull Ups

The pull-up test is considered an effective test for assessing upper body strength—particularly the strength of the upper back, arms, and shoulders. The subject holds the bar with both hands placed squarely (palms forward), slightly wider than shoulder width. They raise themselves until the chin clears the bar, then lower themselves until the elbows are fully extended and in a hanging position, without any swinging motion. In one single trial the maximum number of correctly performed pull-ups was recorded.

(3) Forward Medicine Ball Forward Throw (MBFT) and Medicine Ball Backward Throw (MBBT)

The shot-put backward throw, along with the backward and forward medicine ball throws (MBBT), have been identified as effective assessments for throwing performance and whole-body explosiveness in athletics. Previous reports on the MBFT show high test-retest reliability, with an Intraclass Correlation Coefficient (ICC) of 0.84 (*p* < 0.01), while the MBBT exhibited an ICC of 0.996 (*p* < 0.01). The participants stood at a marked line, aligning their feet in parallel and slightly spaced positions towards the throwing direction. They grasped the ball (refer to Table 2 for its weight) with both hands at the front of their body, executing an integrated physical effort to launch the solid ball forwards or backwards. Each person was given three tries and the maximum throw distance across the measuring tape was document.

(4) Plank Test

The plank test is employed for evaluating core musculature endurance and has yielded exceptional test-retest reliability, with an significant ICC of 0.99. To commence, the participant assumed a prone position on the ground, with support provided by the body utilizing the forearms, elbows, and feet. The elbows remained perpendicular to the ground while lifting the hips, with the body forming a straight line from heel to head. After assuming the “plank” position, the stopwatch was initiated. Participants were asked to perform a single-trial of the plank test, with the test being continued until they could no longer maintain the correct position. The time taken for the test was recorded in seconds.

(5) 2000 m Endurance Run

Various forms of field tests, such as running on an athletic track, have been utilised to evaluate endurance. According to Bunc (1994), the 2000 m run is a practical and straightforward field test that can efficiently assess aerobic capacity. The participants commenced the race upon hearing the signal, which started behind the starting line. They ran the 2 km distance in the shortest feasible time whilst being recorded in increments of 0.01 s. Ultimately, all times were converted into minutes.

(6) Linear Sprint

Rumpf et al. (2011) has previously reported on the reliability of sprint running assessments in youth athletes. In this study, we evaluated the linear sprint ability of athletes by conducting 30 m and 60 m sprints. Athletes were positioned 1 m behind the start line, which triggered the Photoelectric timing system. They could choose between a 3-point start position or a standing start position and sprinted for the designated distance. Each person completed the test twice and rested for 3–5 min between each attempt. The analysis includes the best results from both the 30 m sprint and the 60 m sprint.

The motor competence (MC) of the participants was diagnosed by three generic tests for flexibility (measured by a sit and reach test), agility (measured by the Hexagonal jump test), and balance—measured by a one-legged stance on a balance pad (Donghuateng Sports Apparatus Ltd., Beijing, China; Zhao and Zhao, 2023):

(1) Trunk Flexibility

To assess back and leg flexibility, we used the sit and reach test, known for its high reliability (see also Hoeger et al., 1990; Safrit, 1990).

(2) Hexagon Agility Test

The hexagon test is a reliable measure of agility, with a high test-retest reliability rate (ICC = 0.93, *p* < 0.001). During the test, participants stand with their feet together at the centre of a hexagon, measuring 60 cm per side with 120-degree angles, facing forwards. Upon hearing the start signal, participants then hop clockwise from the center of the hexagon using both legs. After completing three full rounds (18 jumps) along the hexagon, the time is terminated, and the outcome is recorded. Three tests were conducted for each participant, and the swiftest time, measured by 0.01 s, was selected for consequent analysis. A rest period of 3–5 min was allotted between each test.

(3) Balance Test

The balance performance of the athletes was measured by a one-legged stance on a balance pad (balance pad, Donghuateng Sports Apparatus Ltd., Beijing, China).

#### Specific throw performance

To reduce the influence of technology on throwing performance, we used the power-position throwing technique (without run-up, rotation, or glide) to measure the targeted strength of young athletes in shot put and discus (Karampatsos et al., 2011). It is evident that the leg muscles play a significant role in generating force, e.g., given the importance of run-up speed in javelin (Bartlett and Best, 1988) and release velocity in hammer throw (Castaldi et al., 2022) for determining the final throw distance. Javelin athletes utilize a crossover stride technique without a run-up for their throws. Meanwhile, under-16 hammer throwers execute one spin before releasing the hammer, and under-18 hammer throwers perform two spins before releasing the hammer. After warming up, athletes performed their specific discipline as prescribed. They each completed six throws, with the best result being recorded. A break of 3–5 min was given between each testing session. Athletes of varying ages utilised different equipment weights (Table 2).

All assessments were conducted by qualified personnel from renowned sports academies. For the tests with two repetitions, which were not required in the 2-km run, pull-up, and plank tests, test-retest reliability coefficients above r_tt_ > 0.80 were obtained, demonstrating their suitability for individual evaluations. Rigorous training was given to the testers, and standardized testing procedures were followed to minimize errors (Vicente-Rodríguez et al., 2011).

Prior to the testing session, the participants underwent a standardized warm-up routine that included cycling, running, and dynamic stretching exercises. The testing protocols provided by the Chinese Athletics Association were implemented meticulously throughout the testing phase.

### Statistical analysis

All data were analyzed with SPSS (Version 28.0; SPSS Inc., Chicago, IL, United States) and statistical significance was set at *p* ≤ 0.05.

To ensure high concurrent and discriminative validity of anthropometric and motor predictors, the impact of age on test performance should be taken into account—not only in sports settings in general (Höner et al., 2017). Meylanet al. (2010), as well as Höner and Votteler (2016), have studied the youth athletes in both general sports and specifically those in track and field throwing disciplines such as that examined by Figueiredo et al. (2021) and Redondo Castán et al. (2019). The dataset was subjected to univariate ANOVA tests to identify significant differences among the athletes’ age according to the calendar year. As test performances systematically increased with age across the four adolescent age groups (U15 to U18) involved in the study, calendar age (in months) was removed from all predictors using linear regression analysis to prevent confounding effects in subsequent analysis (Hohmann and Siener, 2021). In the linear regression analyses, test results were used as the dependent variable, while age (in months) was used as the independent variable. For the sake of comparing different predictors, the residuals of the regressions were standardised into *z*-values. Therefore, only the age-adjusted *z*-values were used in all subsequent analyses.

The concurrent validity of the three morphological and twelve physical fitness and motor competence measures was evaluated via a bivariate correlation (Pearson) with the throw performance of the discipline-specific standing throw. The discriminative validity of the three morphological and twelve physical fitness and motor competence measures was established by means of a statistical classification of the athletes, using both linear discriminant analysis (DA) and a multilayer perceptron (non-linear neural network; MLP). The chief benefit of utilising the MLP analysis is that it makes minimal assumptions, owing to the learning character of neural networks methods, regarding interrelations within the data. Thus, as opposed to linear discriminant analysis, the multilayer perceptron can identify both linear and non-linear relationships through its iterative learning process. The utilization of both linear DA and non-linear MLP neural networks presents a promising technique to address the issue of talent identification, particularly in elite sports where various talent patterns may result in equivalent performance outcomes among aspiring young athletes. This method has been extensively discussed and supported in previous studies (Philippaerts et al., 2008; Pfeiffer and Hohmann, 2012; Till et al., 2016; Pion et al., 2017).

In the initial stage of both analyses, the four sports were used as the dependent grouping variable, while the test results were utilised as the independent variable set. Next, we analysed the prioritisation of the fifteen independent variables by comparing a group that included a single throw discipline with the other group consisting of the remaining three sports. The objective of this second stage was to identify the most significant anthropometric, physical fitness, and motor competence traits for each of the four track and field throwing events. Moreover, to prioritize the key characteristics of athletes that are specifically relevant to each throwing discipline. The stepwise discriminant analysis was carried out using the “leave-one-out” approach. This means that the categorization of each person was determined using a formula developed from all other (n–1) tests, with the exception of one case reserved for concluding categorization. Correspondingly, the MLP analysis generated three groupings for i) training, ii) validation predictions, and iii) the ultimate grouping of the remaining cases (test sample). Afterwards, the MLP was trained employing 70% of all cases, whereas 20% were expended to authenticate the trained network. Finally, we calculated the classification for the test sample, comprising the remaining 10% of cases. This particular leave-out cross-validation method was reiterated ten times to ensure that each instance was included in the group of athletes eventually classified as hold-outs at least once. To gauge the credibility of this classification method, the percentage of accurate hits from the neural network classification was averaged across the ten trials and the resulting mean value was implemented. Therefore, this process conforms to a 10-fold cross-validation (Siener et al., 2021a). The classification quality of both methods was determined by calculating the proportion of correct assignments, that is, the percentage of athletes correctly identified with their respective throwing disciplines. Contrastingly, the DA considers *a priori* probabilities and a significant portion of the calculation procedure to accommodate for different case numbers in the two groups. On the other hand, in MLP athletes’ classification, equal case numbers in the investigated groups is the norm. Otherwise, the larger categories attract most of the athletes from smaller ones. To prevent this error due to group size, all MLP calculations randomly assigned participants of the remaining three sports, which had comparable group sizes with the investigated single sport group, into a number of groups that matched the size of the single sport group. Therefore, in shot put, we divided the remaining group into two, in discus throw into three, and in the hammer throw analysis into 15 groups. The results obtained from the subgroups were then averaged in these three analyses. Furthermore, an ROC analysis was calculated to illustrate the findings.

## Results

The morphological measures displayed a concurrent validity of low to medium, while the physical fitness and motor competence tests exhibited medium to large concurrent validity in relation to throwing performance. Table 3 reveals that the criterion-oriented validity of the tests differs among the four distinct throwing disciplines.

**TABLE 3:** Correlation values (Pearson, two-sided) between test scores and achieved sport-specific throwing performance to evaluate the concurrent and criterion-oriented validity.

Correlations	Standing shot put performance (*n* = 155)	Spin hammer throw performance (*n* = 35)	Standing discus throw performance (*n* = 116)	Standing javelin throw performance (*n* = 148)
Body height (cm)	0.08	−0.03	0.28^a^	0.08
Body weight (kg)	0.30^b^	0.10	0.33^b^	0.00
BMI	0.15	0.19	0.08	−0.16
Standing long jump (m)	0.36^b^	0.07	0.29^a^	0.45^b^
Triple jump (m)	0.24^a^	0.05	0.06	0.40^b^
Pull-ups (n)	0.40^b^	0.33^c^	0.27^a^	0.29^b^
Plank (s)	0.06	0.24	−0.08	0.14
Sit and reach (cm)	0.36^b^	0.23	0.36^b^	0.32^b^
30-m sprint (s)	−0.30^b^	−0.22	−0.25^a^	−0.40^b^
60-m sprint (s)	−0.46^b^	−0.09	−0.43^b^	−0.47^b^
Medicine ball throw forward (m)	0.71^b^	0.06	0.65^b^	0.40^b^
Medicine ball throw backward (m)	0.74^b^	0.18	0.63^b^	0.48^b^
Hexagonal agility run (3 reps; s)	−0.16^c^	−0.35^c^	−0.24^c^	−0.13
Balance pad one-leg stance (s)	0.37^a^	−0.37^c^	0.15	0.17
2-km ergometer run (min)	−0.04	−0.20	−0.07	−0.05

^a^ *p* < 0.01.

^b^ *p* < 0.001.

c *p* < 0.05.

### Classification by linear discriminant analysis and non-linear neural network

Overall, 67 cases were excluded from the Discriminant Analysis in the four throwing disciplines due to missing data. The remaining total sample size of n = 280 cases was used to calculate the DA, resulting in a 72.9% correct classification rate. In this study, 76.8% of the 99 shot put athletes achieved correct hits, while only 54.5% of the 11 included hammer throw athletes achieved them. Additionally, 50% of the 62 discus athletes achieved correct hits, and 84.3% of the 108 javelin throwers did so. In order to improve accuracy, a cross-validated DA was conducted in a second attempt, with each of the 280 athletes used as a single hold-out case exclusively classified. Using a leave-one-out procedure, this study found that 68.2% of all participants were correctly classified and assigned to their specific throwing discipline as true positives (refer to Table 4). The classification performance was best in the javelin throw, where 82.4% of athletes were assigned correctly, and cross-validation identified only 19 out of 108 athletes as false negatives for another sport (6 for shot put, 2 for hammer throw, and 11 for discus throw). The hammer throw had the highest percentage of false negatives (63.7%), as a result of four youth hammer throwers (36.4%) being wrongly classified under the shot put group and an additional three (27.3%) under the javelin group.

**TABLE 4:** Cross-validated classification of n = 280 single cases of youth track and field athletes from four throwing disciplines on the basis of 13 performance characteristics.

Throwing groups	Predicted throwing discipline			
	Shot put (*n*; percent)	Hammer throw (*n*; percent)	Discus throw (*n*; percent)	Javelin throw (*n*; percent)
Shot put (*n* = 99)	70 (70.7%)	1 (1.0%)	18 (18.2%)	10 (10.1%)
Hammer throw (*n* = 7)	4 (36.4%)	4 (36.4%)	0	3 (27.3%)
Discus throw (*n* = 53)	26 (41.9%)	1 (1.6%)	28 (45.2%)	7 (11.3%)
Javelin throw (*n* = 100)	6 (5.6%)	2 (1.9%)	11 (10.2%)	89 (82.4%)

**FIGURE 3:**
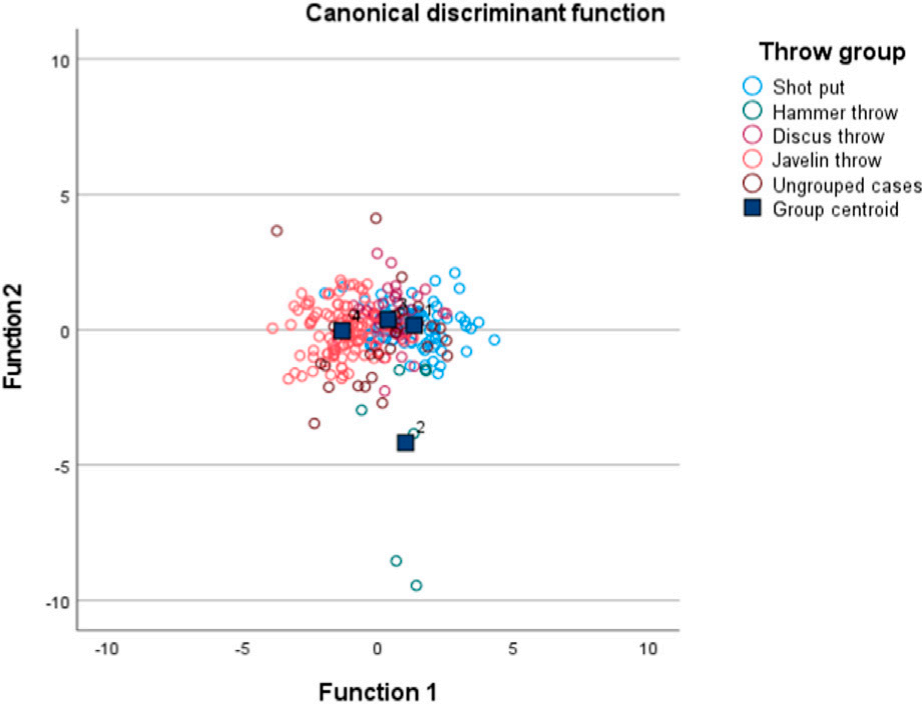
Plot of individual and group differences between the four throw disciplines resulting from three morphological and twelve physical fitness and motor competence tests.

As there were four distinct sports groups, we established three linear discriminant functions. The first two functions accounted for 92.6% of the total variance, and Figure 3 on the X- and Y-axes displays this. The athletes from the four throwing groups are distributed around their respective centroids, which are situated on distinct areas of the plot. Functions at group centroids are identified. Shot put, function 1 = 1.10 and function 2 = −0.21; Hammer throw, function 1 = 0.61 and function 2 = −1.37; Discus throw, function 1 = 0.42 and function 2 = 0.74; and Javelin throw, function 1 = −1.31 and function 2 = −0.09. The first function (Eigenvalue: 1.15) was the most significant, accounting for 78.0 percent of the variance and primarily related to the medicine ball throw (backward). The second function (eigenvalue: 0.22) accounted for 14.6% of the variance and was particularly associated with the performances of jumping and sprinting.

**FIGURE 4:**
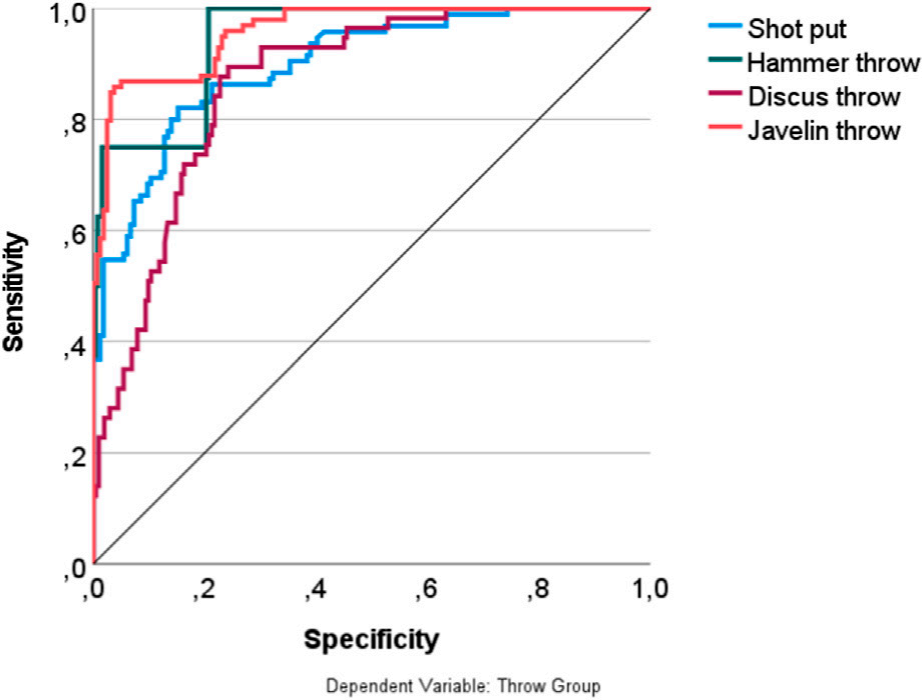
ROC-curves representing the overall quality of the youth athletes’ assignments to the four throw disciplines.

Compared to the results obtained with the DA, the cross-validated MLP attempt resulted in a four percent improvement in predicting the four throwing disciplines. In total, 72.2% of the athletes of the test sample were correctly assigned to their corresponding sport. However, only 10% of the performers were correctly identified in the hammer throw based on their physical fitness and motor competence test data, while a significantly better prediction rate was observed in the other three disciplines. The javelin throw group achieved the best result with 82.9% accurate assignments, followed by the shot put athletes (74.0%) and discus throwers (70.9%). Figure 4 clearly demonstrates the high sensitivity of the non-linear MLP tool, especially in predicting the talent of adolescent discus, javelin throwers, and shot putters based on their physical fitness, motor competence test performances, and morphological features.

### Prioritization of talent characteristics for each throw discipline

Table 5 documents the descriptive statistics of the 15 variables measured in the N = 348 male athletes. The study exhibits the mean age at test for the four groups of throwers, and it reveals no significant difference between them (F3; 285 = 1.80; *p* = 0.147).

**TABLE 5:** Morphological, physical fitness and motor competence performance prerequisites of the athletes from four track and field throwing disciplines.

		N	M	SD	SE	95% Confidence Intervall		Min	Max
						LL	UL		
Age (years)	Shot put	101	15.90	1.00	0.11	15.70	16.10	13.83	17.75
	Hammer throw	16	16.41	0.78	0.19	16.00	16.83	14.83	17.75
	Discus throw	63	16.05	0.96	0.12	15.81	16.29	14.33	17.75
	Javelin throw	109	15.84	1.09	0.10	15.63	16.05	13.92	17.75
Body height (cm)	Shot put	92	181.29	6.50	0.68	179.95	182.64	170.00	200.00
	Hammer throw	9	181.89	6.40	2.13	176.98	186.80	175.00	190.00
	Discus throw	54	184.70	7.43	1.01	182.68	186.73	170.00	198.00
	Javelin throw	101	179.97	5.41	0.54	178.90	181.04	166.00	196.00
Body weight (kg)	Shot put	92	95.86	17.65	1.84	92.21	99.52	61.00	145.00
	Hammer throw	9	104.78	17.97	5.99	90.96	118.59	83.00	125.00
	Discus throw	54	93.39	14.72	2.00	89.3704	97.41	52.00	125.00
	Javelin throw	101	74.56	10.54	1.05	72.48	76.64	52.00	105.00
BMI	Shot put	101	28.56	4.77	0.47	27.62	29.50	10.00	43.20
	Hammer throw	16	28.93	4.026	1.01	26.79	31.08	22.00	34.70
	Discus throw	63	26.88	3.68	0.46	25.95	27.81	17.90	38.00
	Javelin throw	109	23.37	4.75	0.46	22.47	24.27	12.00	42.00
Standing long jump (cm)	Shot put	101	253.04	26.73	2.66	247.76	258.32	179.00	322.00
	Hammer throw	16	252.13	29.00	7.25	236.67	267.58	197.00	290.00
	Discus throw	63	267.03	24.89	3.14	260.76	273.30	200.00	312.00
	Javelin throw	109	259.48	18.97	1.82	255.88	263.08	200.00	300.00
Triple jump (m)	Shot put	101	7.68	1.11	0.11	7.46	7.90	5.20	11.74
	Hammer throw	11	6.92	0.43	0.13	6.63	7.21	6.45	7.51
	Discus throw	62	8.51	1.50	0.19	8.13	8.89	5.90	12.34
	Javelin throw	109	7.77	.73	0.07	7.63	7.91	6.00	11.52
Pull-ups (n)	Shot put	101	6.07	4.52	0.45	5.18	6.96	.00	15.00
	Hammer throw	16	5.50	3.95	0.99	3.40	7.60	.00	14.00
	Discus throw	63	9.00	4.38	0.55	7.90	10.10	.00	16.00
	Javelin throw	109	12.94	8.96	0.86	11.24	14.64	.00	43.00
Plank (s)	Shot put	101	130.32	49.96	4.97	120.45	140.80	39.00	240.00
	Hammer throw	16	235.06	171.16	42.79	143.86	326.27	70.00	660.00
	Discus throw	63	168.10	72.97	9.19	149.72	186.47	60.00	480.00
	Javelin throw	109	172.80	55.79	5.34	162.21	183.39	60.00	310.00
Sit and Reach (cm)	Shot put	101	16.12	6.67	0.66	14.80	17.44	2.00	29.00
	Hammer throw	16	20.25	3.91	0.98	18.17	22.33	14.00	26.00
	Discus throw	63	19.17	4.92	0.62	17.93	20.42	5.00	34.00
	Javelin throw	109	17.69	5.54	0.53	16.64	18.74	1.00	30.00
30 m sprint (s)	Shot put	100	4.51	0.40	0.04	4.44	4.59	3.70	6.00
	Hammer throw	16	4.59	0.31	0.08	4.43	4.76	4.10	5.25
	Discus throw	63	4.26	0.32	0.04	4.18	4.35	3.71	5.03
	Javelin throw	109	4.25	0.38	0.04	4.18	4.32	3.70	5.47
60 m sprint (s)	Shot put	100	8.42	0.81	0.08	8.25	8.58	6.70	11.39
	Hammer throw	11	8.61	0.48	0.14	8.29	8.93	7.97	9.65
	Discus throw	62	7.86	0.62	0.08	7.70	8.01	6.60	9.30
	Javelin throw	108	7.82	0.50	0.05	7.73	7.92	7.00	9.76
Medicine ball throw forward (m)	Shot put	101	12.60	2.23	0.22	12.16	13.04	7.25	18.50
	Hammer throw	16	13.62	1.68	0.42	12.72	14.52	10.80	16.00
	Discus throw	63	13.89	2.11	0.27	13.36	14.42	9.76	20.55
	Javelin throw	109	13.61	2.23	0.21	13.18	14.03	5.00	18.00
Medicine ball throw backward (m)	Shot put	101	15.80	3.36	0.33	15.14	16.47	8.16	21.80
	Hammer throw	11	14.17	2.68	0.81	12.36	15.97	11.64	19.00
	Discus throw	62	16.87	2.42	0.31	16.25	17.48	11.52	21.00
	Javelin throw	109	14.42	2.91	0.28	13.86	14.97	5.00	20.40
Hexagonal agility test (3 reps; s)	Shot put	101	14.39	1.77	0.18	14.04	14.74	11.00	20.00
	Hammer throw	16	13.09	1.27	0.32	12.41	13.77	11.70	16.60
	Discus throw	63	13.08	1.40	0.18	12.72	13.43	9.70	17.00
	Javelin throw	109	14.58	2.68	0.26	14.08	15.09	10.00	24.30
Balance Pad stance (s)	Shot put	101	41.61	17.60	1.83	37.96	45.25	10.00	94.00
	Hammer throw	14	42.50	19.69	5.26	31.13	53.87	19.00	60.00
	Discus throw	62	53.08	18.26	2.32	48.44	57.72	12.00	107.00
	Javelin throw	109	57.65	23.46	2.25	53.20	62.10	20.00	120.00
2 km Ergometer run (min)	Shot put	101	10.42	1.44	0.14	10.13	10.70	8.01	13.05
	Hammer throw	16	10.55	1.43	0.36	9.79	11.31	8.01	12.45
	Discus throw	63	9.90	1.16	0.15	9.61	10.19	8.01	12.00
	Javelin throw	109	9.20	0.89	0.09	9.04	9.37	7.09	12.25

### Shot put

During the stepwise discriminant analysis of anthropometric, physical fitness, and motor competence measures, it was observed that young shot putters demonstrated superior performance in medicine ball backward throw in comparison to all other track and field throwers (refer to Table 5), with a discriminant coefficient of .70. Conversely, shot putters displayed inferior performance in medicine ball forward throw (discriminant coefficient = −.64), 60 m sprint (.38), and plank test (−.44). Based on these five tests, 69.2% of the 101 shot putters that were tested were able to be distinguished from the total group of 188 athletes from other throwing disciplines.

**FIGURE 5:**
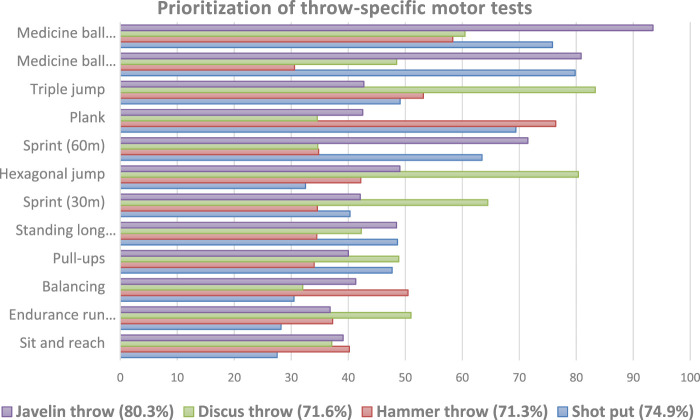
Normalized importance (in percent) of the three morphological and twelve physical fitness and motor competence tests in the non-linear MLP analysis to prioritize each single throw discipline from the remaining total group of all three other sports.

The non-linear MLP analysis (Figure 5) prioritized the importance of the independent variables in classifying the athletes, serving as a validity measure to distinguish between participants of different throwing disciplines based on their anthropometric, physical fitness and motor competence characteristics. The physical fitness tests with the highest relevance in discriminating shot putters from all other throwing athletes were the forward medicine ball throw (importance: 79.8%, despite its negative discrimination against shot putters from other track and field throwers) and an above-average backward throw (importance: 75.8%). Furthermore, a greater BMI (55.5%), slower 60 m sprint times (63.5%), and lower performance in standing long jump (48.6%) were significant contributors in correctly distinguishing 74.9% of shot putters from other throwers.

### Hammer throw

The stepwise DA demonstrated that the hammer throw athletes were set apart from all other youth athletes in the remaining three throwing disciplines mainly due to their exceptional core stability performance in the plank test (discriminant coefficient = 1.13), extended duration of balanced stance on the one-leg pad (.66), and superior agility in the hexagonal jump (−.26). On the contrary, hammer throwers displayed poorer performance in the triple jump (discriminant coefficient = .42) and 2-km running endurance (.34) compared to their peers in other throwing sports. Nonetheless, 44.4 percent of hammer throwers were categorised into their original sporting discipline using the aforementioned five tests.

The non-linear MLP analysis confirmed the DA results, specifically indicating a consistently lower performance in the triple jump (normalized importance: 53.2%). However, the hammer throwers exhibited superior core stability compared to other throwing athletes (plank test importance: 76.4%) and a higher BMI (importance: 56.7%). Overall, the MLP accurately identified 71.3% of the cases included in the test set that was presented to the trained and validated neural network.

### Discus throw

The stepwise DA identified three tests that differentiated this group from other young throwing athletes in the discus throwing discipline. These tests were body height (discriminant coefficient = .64), triple jump distance (discriminant coefficient = .70), and hexagonal jumping agility (discriminant coefficient = −.37). These factors proved superior among all throwers. Despite these significant tests, the DA could only correctly identify 37.0% of the cases.

The MLP analysis found support for the DA results in various areas, such as the superior performance in triple jump (normalized importance: 83.3%) and fast 30-metre sprint (importance: 64.5%), as well as the better results in the medicine ball backward throw test (importance: 60.5%). However, the MLP results also indicated lower hexagonal jumping agility performance (80.4%), which displayed a noticeable deviation from the other young throwing athletes. In brief, the MLP successfully differentiated 71.6% of discus throwers from other participants in throwing events.

### Javelin throw

The stepwise discriminant analysis for the javelin yielded nine significant variables, allowing for the accurate classification of 83% of throwers into their respective sport. Among the discriminating factors were two anthropometric and seven physical fitness and motor competence parameters, distinguishing javelin experts from other participants in track and field throwing disciplines. The medicine ball forward throw displayed the most notable positive contrast compared to all other throwers in the group (discriminant coefficient = −.64). Additionally, an improvement in sprint performances was observed for the 60-m dash (.34), the pull-ups test (−.24), and the 2-km running endurance trial (.24.). Moreover, the lower BMI (.35) was due primarily to a lower body weight (.39). Compared to their counterparts in the other three throwing disciplines, javelin throwers showed lower proficiency in certain aspects of performance. Specifically, they scored less in the backward medicine ball throw (.65), standing long jump (.43), and jumping agility as measured by the hexagonal jump test (−.23).

The non-linear MLP analysis confirmed the DA results to a large extent, as the performance gap between the subject and the total group of other athletes was systematic in the lesser medicine ball backward throw with a normalized importance score of 93.5%. Furthermore, a decreased body weight (85.3%) and lower BMI (56.5%), superior 60-meter sprint performance (71.5%) and above-average agility in the hexagonal test (49.1%) were also significant factors in correctly identifying 80.3% of javelin throw specialists.

## Discussion

The research aimed to differentiate adolescent track and field athletes belonging to four throwing sport disciplines, hailing from more than 30 Chinese elite sports schools. This cohort will likely provide the next-generation of elite senior athletes. The study administered a standardized battery of 15 anthropometric and motor performance measures to a total of 348 athletes. It is important to note that this study employed both linear and non-linear statistical methods simultaneously to determine the key characteristics of throw performance for each of the four throwing disciplines and to validate the findings of each method accordingly. The study found that the generic test battery had a high level of discriminative validity resulting in a correct assignment rate of 58.4% (using linear discriminant analysis) and an even more impressive rate of 74.5% (using the non-linear neural network tool MLP) after cross-validation. Despite the DA achieving a higher classification rate than random assignment, only MLP can be considered a good classification method. The study’s accuracy in distinguishing between adolescent shot putters, hammer, discus, and javelin throwers was of high quality. It is noteworthy that a very homogeneous group of throwing athletes solely from track and field sports was investigated. Roughly three-quarters of the discriminative results were correct.

The value of this discovery is further supported when contrasted with the cross-validated outcomes of Zhao et al. (2019b) who reported 71.3% accurate evaluations in a similar research study at a Chinese leading sports institution in Shanghai that involved a much wider array of sports (basketball, fencing, judo, swimming, table tennis, volleyball). It is advisable to avoid evaluating classification results through cross-validation, as demonstrated by Pion et al.’s (2015) study on nine diverse sports, namely, badminton, basketball, gymnastics, handball, judo, soccer, table tennis, triathlon, and volleyball, in order to achieve better outcomes. The 100% classification achieved by Pion et al. (2014) through a discriminant analysis in the three most homogenous martial arts disciplines, judo, karate and taekwondo, illustrates the need for a cross-check procedure, utilizing either a leave-one-out strategy or a larger hold-out group. Our findings show promise in comparison to Leone et al. (2002) classification rate of 88.0% in a diverse group of figure skaters, swimmers, tennis and volleyball players, and Opstoel et al. (2015) correct hit rate of 85.2% in various sports including ball games, dance, gymnastics, martial arts, racket sports, and swimming athletes. To summarise, our study found that a non-linear neural network accurately classified roughly three-quarters of throwing athletes. However, direct comparison with research groups who only calculated predictive accuracy on the original sample, including all group members, is not possible. In spite of this, we introduced test cases for cross-checking purposes, and our results remain highly satisfactory. This holds true in all four the disciplines and especially in javelin throw, whereas the linear DA and non-linear MLP analyses failed particularly in the identification of hammer throw athletes.

The javelin throw yielded the highest rate of accuracy in the DA, with classification results of 83.0%. The high predictive accuracy is primarily founded on the standing long jump, triple jump, two sprints, and two medicine ball throw tests. This is consistent with Bouhlel et al. (2007) and Schleichardt et al. (2021) findings, which attribute this discovery to the importance of explosive strength skills in this sport. Additionally, body dimensions, such as height and weight, may also play a role. Body weight does not have as significant of an impact in javelin throw compared to discus throw and shot-put disciplines (Carter, 1982; Morrow et al., 1982; Zaras et al., 2013).

As previously mentioned, the anthropometric characteristic of body height is a crucial factor for shot put and discus throw athletes. A tall height allows for a higher take-off and, when combined with longer arms, results in a longer acceleration trajectory of the heavy competition device (Mastalerz and Sadowski, 2022). In addition to comparable anthropometric performance requirements, the performances of throwers from both disciplines in medicine ball throws (forward and backward) as well as their above-average jumping abilities, significantly impact their overall performance in the sports-specific throws.

The linear discriminant analysis had significant deficits in predicting accurately the hammer throw athletes, with about two thirds of them being incorrectly identified. The non-linear multilayer perceptron method displayed superior predictions, but this finding could be attributed to the notably smaller sample size of this sport-specific group. The small number of participants presents a challenge in establishing a distinct sport-specific performance profile, separate from the large number of shot put, discus, and javelin cases. As such, aligning the group sizes used in the MLP procedure is a more appropriate approach than relying on a-priori probability in the DA analysis.

In the past, talent identification for some sports relied mainly on sport-specific tests, which hindered comparisons of results across different sports and impeded drawing conclusions on the feasibility of transferring athletes from donor-to recipient sports. This has become increasingly significant in elite sports during the last 2 decades (Teunissen et al., 2021). Sport-specific tests present a challenge as athletes from one discipline who are not familiar with the techniques and capabilities of another sport cannot reliably take tests that are specific to the other sport. Therefore, it is essential for talent development and transfer processes to incorporate a multidisciplinary combination of anthropometric, motor, psychological, and physiological tests of low to medium specificity. The multifaceted testing protocols enable a comprehensive evaluation of each athlete, as well as comparisons across various sports and transferability between disciplines (Pion, 2015; Pion et al., 2017). Hence, the outcomes of this investigation present significant evidence on the appropriateness of the anthropometric and motor performance exams in identifying young athletes from diverse inter-connected sports categories. Furthermore, there is evidence supporting the effectiveness of using both linear DA and non-linear MLP neural networks as a viable approach to solving the challenge of talent selection and transfer in elite sports. This is particularly relevant when considering the existence of various talent patterns among promising young athletes that may result in equal performance outcomes during later career development stages.

Track and field, in general, and particularly the four throwing disciplines, are commonly regarded as sports that require late specialization (Preckel et al., 2020). The acquisition of technical skills and explosive power that are necessary for these disciplines cannot be attained before the age group of 14–15 years. This is primarily attributed to the significant correlation between biological maturation, chronological age, and the enhancement of physical attributes (Bezuglov et al., 2022), alongside the prolonged instruction required for superior throwing techniques (Kirkeberg et al., 2022). Despite declining age influence in explosive power sports with age, age-related effects persist in senior levels. Hence, an efficient selection or transfer of talent in track and field throwing events is unfeasible prior to this age due to the significant variations in growth spurts, physical traits, and training experiences during puberty that confer greater speed, strength, and size to adolescent athletes (Zhao and Zhao, 2023). The distinctive nature of the throwing motion results in marked differences in fitness characteristics when compared to other sports (Nikolaidis & Son’kin, 2023; Thorland et al., 1981). Therefore, studies of sports-specific characteristics are essential. For example, studies have shown a high correlation between throwing performance and strength in the arms and legs (Bouhlel et al., 2007), as well as explosiveness (Zaras et al., 2021). Thorland et al. (1981) examined junior Olympic athletes and revealed their distinctive anthropometric features. Regarding physical fitness performance in boys of different ages, significant differences were observed in the hexagon agility test, standing long jump, standing triple jump, and MBFT. These results are consistent with previous findings indicating enhanced agility (Fernandez-Fernandez et al., 2023), muscle strength, and power with maturation (Hammami et al., 2016). Furthermore, Zhao and Zhao (2023) presented empirical evidence that male throwing athletes aged 14–18 exhibit a heightened growth rate in agility and lower limb power. Therefore, to further comprehend the precise contribution of adolescent variances in performance and its corresponding morphological and physical attributes, a more in-depth understanding of specific throw-related knowledge on anthropometric and physical performance preconditions is essential. It allows coaches and applied scientists to objectively assess the clear progression of key indicators during the various developmental stages. This, in turn, enables them to design personalised talent selection and transfer initiatives that reflect these changes. It is also crucial to consider the athletes’ developmental opportunities, especially in the four throw disciplines studied in this research. It is essential for adolescent throwing athletes to concentrate on strength, speed, and agility. Coaches must consider the interdependent athletic abilities when selecting and transferring athletes between related disciplines as it could have a transfer effect on subsequent specialisation. Additionally, it should be noted that core fitness attributes discriminate adolescent throwers from peak performance adult athletes due to differences in competition rules for youth throwing athletes. Therefore, for this study, we have selected early and middle adolescence athletes and limited the age range to youth athletes aged between 14 and 17 years.

Our research has some limitations; firstly, due to the high level of specialization of Chinese elite sport school athletes in the technically demanding throwing discipline of hammer throw, we only had a relatively small sample size of 29 athletes, with just eleven cases that had complete data sets. All candidates are subject to rigorous sport-specific selection and training from an early age, suggesting that there were fewer highly specialized hammer throw athletes in the 14–18 year-old male age group studied. Furthermore, the inability of hammer throwers to participate in decathlon events may restrict participation in this discipline.

Secondly, we studied early and middle adolescent throwers aged 14–18 years, so that besides the calendar age the of athletes also the individual status of maturity should be assessed (Meylan et al., 2010; Fernandez-Fernandez et al., 2023). As we did not measure the impact of early and late maturation on the sample of the adolescent throwers this may limit the depth of this study. Thus, further research is needed to clarify the impact of the relative age effect and the biological age for the optimal selection and transfer as well as the athletic development of adolescents throwing athletes.

Another limitation is the exclusive focus on male youth athletes. Additionally, the study focused on solely male athletes due to the complexity of the research and the varying impact of gender-specific athletic composition on sport-specific performance for male and female youth athletes in the four throwing disciplines examined. It is important to note the underrepresentation of female young athletes in Chinese elite sports schools. The limited number of participants in hammer throw and the exclusive focus on male athletes necessitate further investigation into the elite youth sports recruitment processes. Additionally, administering a wider range of motor skills tests, incorporating assessments of coordination and technical proficiency, would have enhanced the evaluation of young athletes.

## Conclusion

The study’s results demonstrate that differences in various multidisciplinary generic anthropometric and (semi-)specific motor performance tests among male athletes aged 14–18 from over 30 Chinese elite sport institutions enable the distinction of more than two-thirds of athletes based on their sport background. This remains true, even when the athletes are utilized as hold-out cases for cross-validation in both linear and non-linear classification methods (DA and MLP). Overall, the talent classification accuracy achieved in Chinese elite youth athletes is consistent with that reported in European studies.

Although our study focused on talent identification and transfer, we found that the performance prerequisites identified for the four track and field throw disciplines can also inform the planning of athletes’ long-term education (Wormhoudt et al., 2018) and the monitoring of seasonal training in young athletes (Young et al., 2015; Bazyler et al., 2017; Sakamoto et al., 2018).

## Data Availability

The original contributions presented in the study are included in the article/supplementary material, further inquiries can be directed to the corresponding author.

## References

[B1] Baker J. Schorer J. Cobley S. (2012). Talent identification and development in sport: international perspectives. London: Routledge.

[B2] Bartlett R. M. Best R. J. (1988). The biomechanics of javelin throwing: a review. J. Sports Sci. 6 (1), 1–38. 10.1080/02640418808729791 3043013

[B3] Bazyler C. D. Mizuguchi S. Harrison A. P. Sato K. Kavanaugh A. A. DeWeese B. H. (2017). Changes in muscle architecture, explosive ability, and track and field throwing performance throughout a competitive season and after a taper. J. Strength Cond. Res. 31 (10), 2785–2793. 10.1519/JSC.0000000000001619 27575250

[B4] Bezuglov E. Shoshorina M. Emanov A. Semenyuk N. Shagiakhmetova L. Cherkashin A. (2022). The relative age effect in the best track and field athletes aged 10 to 15 Years old. Sports (Basel, Switz. 10 (7), 101. 10.3390/sports10070101 PMC9323626 35878112

[B5] Boccia G. Brustio P. R. Moisè P. Franceschi A. La Torre A. Schena F. (2019). Elite national athletes reach their peak performance later than non-elite in sprints and throwing events. J. Sci. Med. Sport 22 (3), 342–347. 10.1016/j.jsams.2018.08.011 30172614

[B6] Boccia G. Cardinale M. Brustio P. R. (2021). Elite junior throwers unlikely to remain at the top level in the senior category. Int. J. Sports Physiology Perform. 16 (9), 1281–1287. 10.1123/ijspp.2020-0699 33647881

[B7] Bouhlel E. Chelly M. S. Tabka Z. Shephard R. (2007). Relationships between maximal anaerobic power of the arms and legs and javelin performance. J. Sports Med. Phys. Fit. 47 (2), 141–146. MID. 17557050

[B8] Buekers M. Borry P. Rowe P. (2015). Talent in sports. Some reflections about the search for future champions. Mov. Sport Sci. - Sci. Mot. (88), 3–12. 10.1051/sm/2014002

[B9] Bullock N. Gulbin J. P. Martin D. T. Ross A. Holland T. Marino F. (2009). Talent identification and deliberate programming in skeleton: ice novice to Winter Olympian in 14 months. J. Sports Sci. 27 (4), 397–404. 10.1080/02640410802549751 19191166

[B10] Bunc V. (1994). A simple method for estimating aerobic fitness. Ergonomics 37 (1), 159–165. 10.1080/00140139408963634 8112271

[B11] Carter J. E. L. (Editor) (1982). Medicine and sport: vol. 16. The Montreal olympic games anthropological project (Karger).

[B12] Castaldi G. M. Borzuola R. Camomilla V. Bergamini E. Vannozzi G. Macaluso A. (2022). Biomechanics of the hammer throw: narrative review. Front. Sports Act. Living 4, 853536. 10.3389/fspor.2022.853536 35434619 PMC9008721

[B13] Caughey R. M. Thomas C. (2022). Variables associated with high school shot put performance. Int. J. Exerc. Sci. 15 (6), 1357–1365. MID. 36582969 10.70252/LGRI2993 PMC9762240

[B14] Cobley S. Schorer J. Baker J. (Editors) (2012). “Identification and development of sport talent: a brief introduction to a growing field of research and practice,” in Talent identification and development in sport. London, New York: Routledge, 1–11.

[B15] Collins R. Collins D. MacNamara A. Jones M. I. (2014). Change of plans: an evaluation of the effectiveness and underlying mechanisms of successful talent transfer. J. Sports Sci. 32 (17), 1621–1630. 10.1080/02640414.2014.908324 24814474

[B16] Douglas A. (2014). “Sifting the sands - talent identification at aspire academy, Qatar,” in Proceedings of the talent identification–identifying champions (Doha: Qatar).

[B17] Fernandez-Fernandez J. Canós-Portalés J. Martínez-Gallego R. Corbi F. Baiget E. (2023). Effects of different maturity status on change of direction performance of youth tennis players. Biol. Sport 40 (3), 867–876. 10.5114/biolsport.2023.121324 37398953 PMC10286620

[B18] Fernandez-Fernández J. Ulbricht A. Ferrauti A. (2014). Fitness testing of tennis players: how valuable is it? Br. J. Sports Med. 48 (1), 22–31. 10.1136/bjsports-2013-093152 PMC3995228 24668375

[B19] Figueiredo L. S. Silva D. Oliveira B. Ferreira A. G. Gantois P. Fonseca F. (2021). Relative age effects in elite Brazilian track and field athletes are modulated by sex, age category, and event type. Motriz: Revista de Educação Física 27. 10.1590/S1980-657420210004621

[B20] Fuchslocher J. Romann M. Rüdisüli L. R. Birrer D. Hollenstein C. (2011). Das Talentselektionsinstrument PISTE: wie die Schweiz nachwuchsathleten auswählt. Leistungssport 41, 22–27.

[B21] Hammami R. Chaouachi A. Makhlouf I. Granacher U. Behm D. G. (2016). Associations between balance and muscle strength, power performance in male youth athletes of different maturity status. Pediatr. Exerc. Sci. 28 (4), 521–534. 10.1123/pes.2015-0231 27046937

[B22] Hartigh R. d. Van Dijk M. Steenbeek H. W. van Geert P. (2016). A dynamical network model to explain the development of excellent human performance. Front. Psychol. 7 (532). 10.3389/fpsyg.2016.00532 PMC4837162 27148140

[B23] Hoare D. (1995). Talent search: the national talent identification and development program: school teacher manual. Sports Coach 18, 24–25.

[B24] Hoeger W. W. Hopkins D. R. Button S. Palmer T. A. (1990). Comparing the sit and reach with the modified sit and reach in measuring flexibility in adolescents. Pediatr. Exerc. Sci. 2 (2), 156–162. 10.1123/pes.2.2.156 39152585

[B25] Hohmann A. Siener M. (2021). Talent identification in youth soccer: prognosis of U17 soccer performance on the basis of general athleticism and talent promotion interventions in second-grade children. Front. Sport Act. Living 3, 625645. Advance online publication. 10.3389/fspor.2021.625645 PMC8212928 34151260

[B74] Hohmann A. Seidel I. (2003). Scientific aspects of talent development. Int. J. Phys. Educ. 40 (1), 9–20.

[B26] Höner O. Leyhr D. Kelava A. (2017). The influence of speed abilities and technical skills in early adolescence on adult success in soccer: a long-term prospective analysis using ANOVA and SEM approaches. PLOS ONE 12 (8), e0182211. 10.1371/journal.pone.0182211 28806410 PMC5555567

[B27] Höner O. Votteler A. (2016). Prognostic relevance of motor talent predictors in early adolescence: a group- and individual-based evaluation considering different levels of achievement in youth football. J. Sports Sci. 34 (24), 2269–2278. 10.1080/02640414.2016.1177658 27148644

[B28] Horst F. Janssen D. Beckmann H. Schöllhorn W. I. (2020). Can individual movement characteristics across different throwing disciplines Be identified in high-performance decathletes? Front. Psychol. 11, 2262. 10.3389/fpsyg.2020.02262 33041901 PMC7530176

[B29] Jha P. Nuhmani S. Kapoor G. Al Muslem W. H. Joseph R. Kachanathu S. J. (2022). Efficacy of core stability training on upper extremity performance in collegiate athletes. J. Musculoskelet. Neuronal Interact. 22 (4), 498–503. MID. 36458387 PMC9716297

[B30] Karampatsos G. Terzis G. Georgiadis G. (2011). Muscular strength, neuromuscular activation and performance in discus throwers. J. Phys. Educ. Sport 11, 369. http://efsupit.ro/images/stories/imgs/jpes/2011/12/1art57.pdf.

[B31] Kim H. Lee Y. Shin I. Kim K. Moon J. (2014). Effects of 8 weeks' specific physical training on the rotator cuff muscle strength and technique of javelin throwers. J. Phys. Ther. Sci. 26 (10), 1553–1556. 10.1589/jpts.26.1553 25364111 PMC4210396

[B32] Kirkeberg A. Roaas T. V. Gundersen H. Dalen T. (2022). Relative age effect among the best Norwegian track and field athletes of all time: comparisons of explosive and endurance events. Front. Psychol. 13, 858095. 10.3389/fpsyg.2022.858095 35903745 PMC9315261

[B33] Leone M. Lariviere G. Comtois A. S. (2002). Discriminant analysis of anthropometric and biomotor variables among elite adolescent female athletes in four sports. J. Sports Sci. 20 (6), 443–449. 10.1080/02640410252925116 12137174

[B34] Łysoń-Uklańska B. Błażkiewicz M. Kwacz M. Wit A. (2021). Muscle force patterns in lower extremity muscles for elite discus throwers, javelin throwers and shot-putters - a case study. J. Hum. Kinet. 78, 5–14. 10.2478/hukin-2021-0026 34025859 PMC8120960

[B35] MacNamara Á. Collins D. (2015). Second chances: investigating athletes' experiences of talent transfer. PloS One 10 (11), e0143592. 10.1371/journal.pone.0143592 26600303 PMC4658000

[B36] Mastalerz A. Sadowski J. (2022). Variability of performance and kinematics of different shot put techniques in elite and sub-elite athletes-A preliminary study. Int. J. Environ. Res. Public Health 19 (3), 1751. 10.3390/ijerph19031751 35162774 PMC8835003

[B37] Meylan C. Cronin J. Oliver J. Hughes M. (2010). Talent identification in soccer: the role of maturity status on physical, physiological and technical characteristics. Int. J. Sports Sci. Coach. 5 (4), 571–592. 10.1260/1747-9541.5.4.571

[B38] Morrow J. R. Disch J. G. Ward P. E. Donovan T. J. Katch F. I. Katch V. L. (1982). Anthropometric, strength, and performance characteristics of American world class throwers. J. Sports Med. Phys. Fit. 22 (1), 73–79. 7132320

[B39] Nikolaidis P. T. Son'kin V. D. (2023). Sports Physiology in adolescent track-and-field athletes: a narrative review. Open Access J. Sports Med. 14, 59–68. 10.2147/OAJSM.S417612 37404686 PMC10317547

[B40] Norton K. I. (2018). Standards for anthropometry assessment. Kinanthropometry Exerc. Physiol., 68–137. 10.4324/9781315385662-4

[B41] Okada T. Huxel K. C. Nesser T. W. (2011). Relationship between core stability, functional movement, and performance. J. Strength Cond. Res. 25 (1), 252–261. 10.1519/JSC.0b013e3181b22b3e 20179652

[B42] Opstoel K. Pion J. Elferink-Gemser M. Hartman E. Willemse B. Philippaerts R. (2015). Anthropometric characteristics, physical fitness and motor coordination of 9 to 11 Year old children participating in a wide range of sports. PLOS ONE 10 (5), e0126282. 10.1371/journal.pone.0126282 25978313 PMC4433213

[B43] Pfeiffer M. Hohmann A. (2012). Applications of neural networks in training science. Hum. Mov. Sci. 31 (2), 344–359. 10.1016/j.humov.2010.11.004 21315468

[B44] Philippaerts R. M. Coutts A. Vaeyens R (2001). “Physiological Perspectives on the Identification and Development of Talented Performers in Sport,” in Talent Identification and Development. The Search for Sporting Excellence. Editors Fisher R. Bailey R. (Berlin: ICSSPE), 49–67.

[B45] Pion J. (2015). The Flemish sports compass: from sports orientation to elite performance prediction.

[B46] Pion J. Fransen R. Lenoir M. Segers V. (2014). The value of non-sport-specific characteristics for talent orientation in young male judo, karate and taekwondo athletes. Arch. Budo 10, 147–152.

[B47] Pion J. Hohmann A. Liu T. Lenoir M. Segers V. (2017). Predictive models reduce talent development costs in female gymnastics. J. Sports Sci. 35 (8), 806–811. 10.1080/02640414.2016.1192669 27267568

[B48] Pion J. Segers V. Fransen J. Debuyck G. Deprez D. Haerens L. (2015). Generic anthropometric and performance characteristics among elite adolescent boys in nine different sports. Eur. J. Sport Sci. 15 (5), 357–366. 10.1080/17461391.2014.944875 25143133

[B49] Preckel F. Golle J. Grabner R. Jarvin L. Kozbelt A. Müllensiefen D. (2020). Talent development in achievement domains: a psychological framework for within- and cross-domain research. Perspect. Psychol. Sci. A J. Assoc. Psychol. Sci. 15 (3), 691–722. 10.1177/1745691619895030 32196409

[B50] Redondo Castán J. C. Fernández-Martínez E. Izquierdo J. M. (2019). Efecto de la edad relativa en las disciplinas de lanzamientos de los participantes españoles en el plan nacional de tecnificación de atletismo. Cuad. De. Psicol. Del Deporte 19 (3), 156–167. 10.6018/cpd.378391

[B51] Rumpf M. C. Cronin J. B. Oliver J. L. Hughes M. (2011). Assessing youth sprint ability-methodological issues, reliability and performance data. Pediatr. Exerc. Sci. 23 (4), 442–467. 10.1123/pes.23.4.442 22109773

[B52] Safrit M. J. (1990). The validity and reliability of fitness tests for children: a review. Pediatr. Exerc. Sci. 2 (1), 9–28. 10.1123/pes.2.1.9 39152574

[B53] Sakamoto A. Kuroda A. Sinclair P. J. Naito H. Sakuma K. (2018). The effectiveness of bench press training with or without throws on strength and shot put distance of competitive university athletes. Eur. J. Appl. Physiology 118 (9), 1821–1830. 10.1007/s00421-018-3917-9 29931495

[B54] Schleichardt A. Badura M. Lehmann F. Ueberschär O. (2021). Comparison of force-velocity profiles of the leg-extensors for elite athletes in the throwing events relating to gender, age and event. Sports Biomech. 20 (6), 720–736. 10.1080/14763141.2019.1598479 31132026

[B55] Siener M. Faber I. Hohmann A. (2021a). Prognostic validity of statistical prediction methods used for talent identification in youth tennis players based on motor abilities. Appl. Sci. 11 (15), 7051. 10.3390/app11157051

[B56] Siener M. Ferrauti A. Hohmann A. (2021b). Talent orientation: the impact of motor abilities on future success in tennis. Int. J. Racket Sports Sci. 3 (2), 26–38. 10.30827/Digibug.73876

[B57] Siener M. Hohmann A. (2019). Talent orientation: the impact of motor abilities on future success in table tennis. Ger. J. Exerc. Sport Res. 49 (3), 232–243. 10.1007/s12662-019-00594-1

[B58] Stone M. H. Sanborn K. O'Bryant H. S. Hartman M. Stone M. E. Proulx C. (2003). Maximum strength-power-performance relationships in collegiate throwers. J. Strength Cond. Res. 17 (4), 739–745. 10.1519/1533-4287(2003)017<0739:msrict>2.0.co;2 14636111

[B59] Terzis G. Georgiadis G. Vassiliadou E. Manta P. (2003). Relationship between shot put performance and triceps brachii fiber type composition and power production. Eur. J. Appl. Physiology 90 (1-2), 10–15. 10.1007/s00421-003-0847-x 12768426

[B60] Teunissen J. W. A. Welle S. S. Platvoet S. S. Faber I. Pion J. Lenoir M. (2021). Similarities and differences between sports subserving systematic talent transfer and development: the case of paddle sports. J. Sci. Med. Sport 24 (2), 200–205. 10.1016/j.jsams.2020.09.005 32972845

[B61] Thorland W. G. Johnson G. O. Fagot T. G. Tharp G. D. Hammer R. W. (1981). Body composition and somatotype characteristics of junior Olympic athletes. Med. Sci. Sports Exerc. 13 (5), 332–338. 10.1249/00005768-198105000-00012 7321833

[B62] Till K. Jones B. L. Cobley S. Morley D. O'Hara J. Chapman C. (2016). Identifying talent in youth sport: a novel methodology using higher-dimensional analysis. PLOS ONE 11 (5), e0155047. 10.1371/journal.pone.0155047 27224653 PMC4880304

[B63] Unnithan V. White J. Georgiou A. Iga J. Drust B. (2012). Talent identification in youth soccer. J. Sports Sci. 30 (15), 1719–1726. 10.1080/02640414.2012.731515 23046427

[B64] Vicente-Rodríguez G. Rey-López J. P. Ruíz J. R. Jiménez-Pavón D. Bergman P. Ciarapica D. (2011). Interrater reliability and time measurement validity of speed-agility field tests in adolescents. J. Strength Cond. Res. 25 (7), 2059–2063. 10.1519/JSC.0b013e3181e742fe 21499136

[B65] Williams A. M. Franks A. M. (1998). Talent identification in soccer. Sports Exerc. Inj. (4), 159–165.

[B66] Williams A. M. Reilly T. (2000). Talent identification and development in soccer. J. Sports Sci. 18 (9), 657–667. 10.1080/02640410050120041 11043892

[B67] Wormhoudt R. Savelsbergh G. Teunissen J. W. Davids K. (2018). Athletic skills model: optimizing talent development through movement education. London: Routledge.

[B68] Young K. P. Haff G. G. Newton R. U. Gabbett T. J. Sheppard J. M. (2015). Assessment and monitoring of ballistic and maximal upper-body strength qualities in athletes. Int. J. Sports Physiology Perform. 10 (2), 232–237. 10.1123/ijspp.2014-0073 25115146

[B69] Zaras N. Spengos K. Methenitis S. Papadopoulos C. Karampatsos G. Georgiadis G. (2013). Effects of strength vs. Ballistic-power training on throwing performance. J. Sports Sci. Med. 12 (1), 130–137. MID. 24149736 PMC3761775

[B70] Zaras N. Stasinaki A.-N. Terzis G. (2021). Biological determinants of track and field throwing performance. J. Funct. Morphol. Kinesiol. 6 (2), 40. 10.3390/jfmk6020040 34067149 PMC8163003

[B71] Zhao K. Hohmann A. Chang Y. Zhang B. Pion J. Gao B. (2019a). Physiological, anthropometric, and motor characteristics of elite Chinese youth athletes from six different sports. Front. Physiology 10, 405. Article 405. 10.3389/fphys.2019.00405 PMC6499036 31105576

[B72] Zhao K. Hohmann A. Gao B. (2019b). Talent identification in elite youth sports. Med. Sci. Sports Exerc. 51 (6S), 623–624. 10.1249/01.mss.0000562364.45703.56

[B73] Zhao Y. Zhao K. (2023). Anthropometric measurements, physical fitness performance and specific throwing strength in adolescent track-and-field throwers: age, sex and sport discipline. Appl. Sci. 13, 10118. 10.3390/app131810118

